# Interleukin-6 is a significant predictor of radiographic knee osteoarthritis: The Chingford study

**DOI:** 10.1002/art.24598

**Published:** 2009-07

**Authors:** Gregory Livshits, Guangju Zhai, Deborah J Hart, Bernet S Kato, Huizhong Wang, Frances M K Williams, Tim D Spector

**Affiliations:** 1 King's College LondonLondon, UK; 2 King's College LondonLondon, UK; 3 305 Hospital of the Chinese PLABeijing, China; 4 Tel Aviv University, Tel AvivIsrael

## Abstract

**Objective:**

There is a great need for identification of biomarkers that could improve the prediction of early osteoarthritis (OA). We undertook this study to determine whether circulating levels of interleukin-6 (IL-6), tumor necrosis factor α (TNFα), and C-reactive protein (CRP) can serve as useful markers of radiographic knee OA (RKOA) in a normal human population.

**Methods:**

RKOA data were obtained from the cohort of the Chingford Study, a prospective population-based study of healthy, middle-aged British women. The RKOA-affected status of the subjects was assessed using the Kellgren/Lawrence (K/L) grade as determined on radiographs obtained at baseline (n = 908) and at 10 years and 15 years thereafter. Serum levels of CRP, IL-6, and TNFα were assayed at 5, 8, and 15 years, using high-sensitivity commercial assays. A K/L grade of ≥2 in either knee was used as the outcome measure. Statistical analyses included analysis of variance for repeated measurements and logistic regression models, together with longitudinal modeling of dichotomous responses.

**Results:**

During 15 years of followup, the prevalence of RKOA (K/L grade ≥2) increased from 14.7% to 48.7% (*P* < 0.00001 versus baseline). The body mass index (BMI) and circulating levels of CRP and IL-6 were consistently and significantly higher in subjects diagnosed as having RKOA. When multiple logistic regression was applied to the data, the variables of older age (*P* = 3.93 × 10^−5^), higher BMI at baseline (*P* = 0.0003), and increased levels of IL-6 at year 5 (*P* = 0.0129) were determined to be independent predictors of the appearance of RKOA at year 10. The results were fully confirmed using longitudinal modeling of repeated measurements of the data obtained at 3 visits. The odds ratio for RKOA in subjects whose IL-6 levels were in the fourth quartile of increasing levels (versus the first quartile) was 2.74 (95% confidence interval 1.94–3.87).

**Conclusion:**

This followup study showed that individuals were more likely to be diagnosed as having RKOA if they had a higher BMI and increased circulating levels of IL-6. These results should stimulate more work on IL-6 as a potential therapeutic target.

Osteoarthritis (OA) is the most common form of arthritis and can result in substantial morbidity and disability in the elderly (1). It is known that OA imposes a great economic burden on modern society (2, 3). Risk assessment or diagnosis at the early stages of the disease, coupled with the development of preventive or therapeutic interventions, could substantially improve the quality of life for the elderly and reduce health care costs. The development of preventive strategies and early-stage interventions for OA is likely to depend on identification of the biologic mechanisms and biomarkers that underlie progressive deterioration of joint structure and function. However, there are still no commonly accepted and reliable biomarkers for predicting the development and progression of OA and for distinguishing mild, age-related disease from the rarer form of aggressive, rapidly progressive disease.

Since OA is a heterogeneous and multifactorial process of joint degeneration, various mechanisms may be involved in its development. Inflammation is potentially a key mechanism that appears to act through alteration of cytokine profiles, which occurs secondary to aging of the immune system or obesity (4–7). Interleukin-1β (IL-1β) and tumor necrosis factor α (TNFα) are of special interest in the elderly, because both cytokines induce production of IL-6 and because they have profound effects on body metabolism, body composition, and the acute-phase response (8–10), all of which are altered with increasing age. TNFα has been shown to regulate energy expenditure in humans during inflammation and is associated with low lean body mass, which is an important marker of physiologic status and a major predictor of survival, strength, and functional status in the elderly (11).

The role of IL-6 in inflammation differs from that of TNFα. The levels of IL-6 are elevated in a variety of inflammatory conditions (12) and in bone resorption (13). However, IL-6 does not cause symptoms of inflammation when infused at high doses, but rather it suppresses the synthesis of other inflammatory cytokines (14) and causes hepatic production of acute-phase proteins such as C-reactive protein (CRP). Increased circulating levels of CRP and IL-6 have been found to be predictors of reduced physical mobility (15) and incident mobility limitation (4) in the elderly. Moreover, there is a growing body of evidence, obtained mostly from cross-sectional studies, suggesting that their levels, especially those of CRP, are elevated in OA (16–18), although results in recent reports have been contradictory (19). To the best of our knowledge, the contribution of inflammatory factors such as IL-6 and TNFα to the severity and incidence of OA has not been examined systematically; in particular, the course of these factors longitudinally over multiple time points has yet to be defined.

The major aim of this study was to explore whether the circulating levels of 3 key inflammation markers, CRP, IL-6, and TNFα, are independently associated with radiographic knee OA (RKOA) in a large cohort of middle-aged European women who were followed up for 15 years. We were also interested in determining whether the effect of these markers is independent of the body mass index (BMI), a known significant predictor of OA (e.g., see ref.^19^).

## PATIENTS AND METHODS

### Study cohort

The participants of this study were white women, and the data for this cohort were collected within the framework of the Chingford Study. This is a well-established, prospective population-based study of middle-aged British women who have been evaluated intensively for OA and osteoporosis since 1989. The cohort originally consisted of 1,003 women ages 45–64 years, selected through general practitioner records. All subjects were recruited from a sample of 1,353 women as part of a health trial; to avoid selection bias, the subjects were not aware of the research into OA. There were no inclusion/exclusion criteria other than age. The cohort has been followed up biannually, and many clinical, anthropometric, psychosocial, radiologic, and metabolic variables have been recorded on at least 2 time points (20, 21). The Chingford Study is currently approaching its twentieth year and has retained ∼65% of the cohort, with a current age range of 60–82 years. All subjects still participating in the Chingford Study were reexamined for the present study in Chingford Hospital in North-East London.

The radiologic and biochemical factors relevant to the present study included RKOA status obtained from radiographs of both knees, and the serum concentrations of 3 inflammatory cytokines, namely, CRP, IL-6, and TNFα. The radiologic examinations were carried out at year 1 (entry examination [baseline]) and at year 10 and year 15 thereafter. Blood samples were obtained at years 5, 8, and 15. Anthropometric assessments, including determination of the BMI, were carried out at all visits. Table 1 provides details of the study sample for each variable and at each time point during the 15 years of followup.

**Table 1:** Basic descriptive statistics of the study sample by variable and visit*

				Skewness†		
Variable, visit	No. of subjects	Mean ± SD	Median (minimum–maximum)	Mean (SEM)	Ln_dst	R for age‡
Body mass index, kg/m^2^						
Year 1	1,002	25.585 ± 4.251	24.850 (16.810–44.540)	1.201 (0.077)	0.066	0.102
Year 10	810	26.784 ± 4.726	26.300 (17.000–59.400)	1.159 (0.085)	0.435	NS
Year 15	638	27.213 ± 4.809	26.587 (16.344–46.991)	0.652 (0.096)	0.145	NS
Serum cytokine levels						
hsCRP, mg/liter						
Year 5	417	0.290 ± 0.383	0.164 (3.850–0.020)	4.109 (0.120)	0.087	NS
Year 8	474	0.263 ± 0.997	0.143 (21.200–0.012)	19.703 (0.112)	0.346	0.096
Year 15	319	0.300 ± 0.404	0.203 (5.010–0.020)	6.607 (0.137)	−0.026	NS
IL-6, pg/ml						
Year 5	429	2.034 ± 3.690	0.976 (24.889–0.185)	4.475 (0.118)	1.055	0.215
Year 8	473	3.002 ± 4.319	1.560 (30.228–0.307)	3.654 (0.112)	0.870	0.139
Year 15	322	1.935 ± 2.386	1.267 (20.028–0.162)	4.451 (0.136)	0.495	0.192
TNFα, pg/ml						
Year 5	430	8.367 ± 17.333	3.375 (173.798–0.642)	5.379 (0.118)	0.904	0.151
Year 8	473	3.596 ± 5.420	1.851 (48.061–0.672)	4.488 (0.112)	1.399	0.172
Year 15	322	1.496 ± 0.848	1.192 (7.136–0.138)	2.065 (0.136)	0.305	NS
Age, years						
Year 1	1,003	54.68 ± 6.02	54.256 (44–68)	–	–	–
Year 10	1,002	64.46 ± 6.16	64.080 (53–78)	–	–	–
Year 15	645	69.12 ± 5.81	68.523 (60–82)	–	–	–

* NS = not significant (*P* > 0.05); hsCRP = high-sensitivity C-reactive protein; IL-6 = interleukin-6; TNFα = tumor necrosis factor α.

† Mean (SEM) values are the estimate of skewness of the original, nontransformed data, while the Ln_dst represents the results of the test for normality of the distribution of the natural logarithm–transformed data (new estimate of skewness).

‡ Values are the root square multiple-determination correlation coefficient for age or, if significantly associated, age-squared.

### Identification of RKOA

Anteroposterior extended-view weight-bearing radiographs of the knees were obtained at baseline and at 10 years and 15 years thereafter. Views were standardized for position, with the backs of the knees in contact with the cassette, the patella centralized over the lower end of the femur, and the beam centered 2.5 cm below the apex of the patella, with a tube-to-film distance of 100 cm. Radiographs were read by examiners (DJH and TDS) who were blinded to the clinical information, with the readings checked against an atlas of radiographic features to determine a global Kellgren/Lawrence (K/L) grade of RKOA (scale 0–4) (22) and to evaluate individual features of osteophytes and joint space narrowing (scale 0–3 for both).

A case of RKOA was defined as a K/L grade of ≥2 in either knee. Pairs of knee films were read by a single investigator (DJH or TDS). Reproducibility of this method showed that intraobserver agreement for side-by-side readings was high (κ = 0.88), and reproducibility for detecting change in longitudinal radiographs was also relatively high (κ = 0.79). We found little difference in the reproducibility between blinded readings (intraobserver κ = 0.83, longitudinal κ = 0.70) and side-by-side readings, so the main radiographic analysis was performed with baseline and followup films read side-by-side. In the statistical analysis, we examined the association of the predictor variables with a dichotomous variable (yes versus no) indicating the RKOA-affected status of individuals, with 1 indicating affected (i.e., K/L grade ≥2) and 0 indicating unaffected (i.e., K/L grade <2).

### Measurement of cytokine levels

Since blood samples were not available for the entire cohort, some data on cytokine levels were missing; the corresponding sample sizes for each variable are given in Table 1. After subjects fasted overnight, blood samples were obtained by venepuncture, and the cells were removed by centrifugation. Serum aliquots were stored at −70°C until analyzed. Serum CRP levels were determined with a high-sensitivity nephelometric method using the Beckman Image Immunochemistry system (Beckman Instruments, Fullerton, CA), which has a minimum level of detection of 0.2 mg/liter. Serum levels of TNFα were measured using a high-sensitivity commercial enzyme-linked immunosorbent assay (ELISA) with an alkaline phosphatase signal amplification system for TNFα (Quantikine High-Sensitivity ELISA, catalog no. SSTA00D, lot no. 246936; R&D Systems, Minneapolis, MN). Serum levels of IL-6 were measured using an ultrasensitive ELISA kit (hIL-6 Ultra-Sensitivity ELISA, catalog no. KHC0064, lot no. S070617D; BioSource, Nivelles, Belgium). All measurements were carried out in accordance with the manufacturers' instructions and were implemented using the microplate reader Opsys MR (Dynex, Alexandria, VA). The minimum levels of detection were 0.038 pg/ml and <0.104 pg/ml for TNFα and IL-6, respectively. The intra- and interassay coefficients of variation for measurements of CRP, IL-6, and TNFα were 2.6%, 4.6%, and 5.3%, respectively, and 3.1%, 5.4, and 7.5%, respectively.

### Statistical analysis

Basic statistical analysis of the data obtained at each visit included analysis of the distribution properties of the variables, as well as correlation analyses to assess possible associations between each quantitative trait (BMI and cytokine levels) and age and between traits. Specifically, we examined the pairwise correlations between BMI and levels of various cytokines within each year and between the repeated measurements of the same variable (e.g., IL-6 at year 5 and IL-6 at year 8). Comparison of baseline measurements of the continuous traits (BMI and cytokine levels) with followup measurements was carried out using paired *t*-test and Friedman's nonparametric repeated-measures comparison test, to ensure that deviations from normality did not bias the parameter estimates, with nonindependence of the measurements taken into account. Friedman's test was also used to compare the prevalence of RKOA among subjects at baseline (year 1) and in the repeated examinations at years 10 and 15 of the followup period. Cochran's Q test for matched sets of frequencies was also implemented, to ensure the reliability of the observed significant differences.

For preliminary recognition of the potential predictor variables for RKOA, the relationship between each variable and RKOA was tested using a series of univariate analyses of variance (ANOVAs). Specifically, we compared BMI and cytokine levels by RKOA-affected status and at each visit (years 1, 10, and 15). A similar procedure was then applied to the combined data from 3 visits for each independent variable separately, with simultaneous adjustment of the significance level (*P* values) for repeated measurements. Specifically, we compared BMI values collected at years 1, 10, and 15 and cytokine levels collected at years 5, 8, and 15 in individuals with and those without RKOA. All of these statistical analyses were conducted using the Statistica package, version 7 (StatSoft, Minneapolis, MN).

The relative effect of the selected potential predictors of the risk of development of RKOA was next examined using logistic regression analysis with multiple predictor variables. This was carried out in 2 stages. First, the dichotomous variable for RKOA-affected status (affected [K/L grade ≥2] versus unaffected [K/L grade <2]) at year 10 was analyzed by standard logistic regression in a model that included quantitative (continuous) variables (age and BMI at entry examination, and cytokine levels measured at year 5) as predictors of risk probability (as well as the odds ratio [OR] with 95% confidence interval [95% CI]). In addition, longitudinal modeling of the dichotomous responses was undertaken using models implemented with the Stata statistical package (version 10; StataCorp, College Station, TX). Similar to the above-described logistic regression analysis, the longitudinal models also tested the risk of RKOA as a function of the predictor variables of interest. The data consisted of longitudinal dichotomous responses, with measurements at years 1, 10, and 15, and data were nested in subjects, which meant that there was dependence among the responses within the same subject.

In addition, a mixed-effects logistic model was used to evaluate the relationship across time (at years 1, 10, and 15) between the presence of RKOA (i.e., K/L grade ≥2) and the continuous predictor variables, including age and BMI, which were collected during the same years as the RKOA data, and the levels of inflammatory cytokines, which were selected in the univariate analyses and assayed at years 5, 8, and 15. This type of logistic model accounts for the intraindividual dependence both in the RKOA data and in the predictor variables (for further details, see ref.^23^).

## RESULTS

### Descriptive statistics

The sample characteristics, including the sample sizes for each trait determined at each visit as well as the levels of circulating cytokines and BMI, are provided in Table 1. Not all individuals whose radiographic and BMI data were available could be assayed for cytokine levels. Initial analyses revealed that the data on cytokine levels were not distributed normally, in that values showed significant skewness to the right. These data were therefore subjected to adjustment using natural logarithm transformation, which improved the data distribution substantially (Table 1).

Nine hundred eight individuals with complete data on the K/L grade of RKOA were available at the entry examination, while 2 subjects were lost to followup by year 10, and 293 subjects were lost to followup by year 15. The main reasons for the loss to followup by year 15 included mortality unrelated to RKOA (99 individuals), withdrew from the study (85 individuals), moved to a different address (75 individuals), and unknown reasons (36 individuals). At baseline, 15.3% of subjects reported having knee pain. During followup, the prevalence of RKOA in either knee increased from 14.7% to 37.4% by year 10, and to 48.7% by year 15. The observed change in RKOA prevalence over the 3 visits was highly statistically significant (χ^2^ = 330.1, 2 df; *P* < 0.00001 versus baseline, by Friedman's test). The prevalence of RKOA in each knee, as assessed by K/L grade, was comparable, with a slight excess in the right knee compared with the left knee, with prevalences at baseline, year 10, and year 15 of 11.0% versus 8.8%, 30.9% versus 25.6%, and 42.1% versus 36.3%, respectively. These differences in RKOA prevalence between knees appeared to be statistically significant (*P* = 0.042–0.001 at each visit separately, by Friedman's test and, especially, by Cochran's Q test; *P* < 0.00001 for the 3 visits combined).

Circulating levels of each of the 3 cytokines were within the ranges expected for a normal population. The correlations of the circulating cytokine levels with age were all low and, in some instances, statistically nonsignificant (Table 1). There were no significant correlations between the individual measurements of TNFα at different visits; however, mean TNFα levels clearly decreased from year 5 to year 15 (*P* < 0.001) (Table 1). The mean values for CRP and IL-6 showed no clear trend with each followup visit. When individual serum concentrations of CRP and IL-6 were compared between the years, we found low correlations between the IL-6 levels (r = 0.22–0.24, *P* < 0.01) and moderate correlations between the CRP concentrations (r = 0.46–0.48, *P* < 0.001). Adjusted for age, IL-6 levels correlated significantly with CRP concentrations in all possible pairwise comparisons, with correlations ranging from 0.22 to 0.48 (*P* < 0.01). Of note, there was very little bias resulting from withdrawal from the study, since age, IL-6 levels, and RKOA prevalence rates among those who withdrew were all comparable with the values in the total sample, specifically for RKOA prevalence at baseline (*P* = 0.26) and for the total assayed level of IL-6 at year 5 (*P* = 0.94).

### Association between RKOA status and circulating biomarker levels

**Figure 1:**
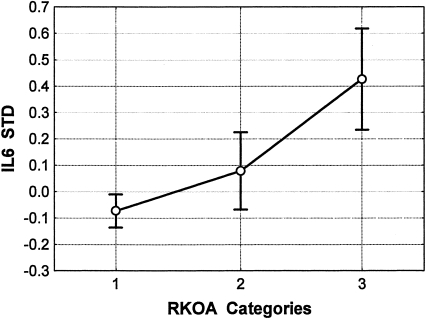
Mean interleukin-6 (IL-6) levels standardized for visit-specific mean and variance (IL6 STD) in 3 radiographic knee osteoarthritis (RKOA) categories: 1 = unaffected (Kellgren/Lawrence [K/L] grade <2), 2 = initial stage of RKOA (K/L grade 2), and 3 = severe RKOA (K/L grade >2). Bars show the 95% confidence intervals. The unadjusted sample sizes were 931, 174, and 101 individuals in RKOA categories 1, 2, and 3, respectively.

The mean age, BMI measurements, and IL-6 levels were consistently higher in RKOA-affected individuals than in unaffected individuals, as determined by univariate ANOVAs (Table 2). When the respective data from the 3 visits (for BMI and IL-6 measurements separately) were combined and the implemented ANOVAs were corrected for repeated measurements, the differences between RKOA-affected and unaffected individuals were significant (*P* < 0.001) for both variables. In particular, the circulating levels of IL-6 were substantially higher in individuals with severe RKOA (defined as a K/L grade >2 in either or both knees) (Figure 1). CRP levels were also consistently higher in affected individuals, although the differences in CRP levels between affected and unaffected individuals did not reach statistical significance in 4 of 9 comparisons. However, when the data from the 3 visits were combined and corrected for repeated measurements, there was a significant trend toward higher levels of CRP in affected individuals (*P* = 0.002 versus unaffected).

**Table 2:** Comparison of the natural logarithm–transformed cytokine levels by radiographic knee osteoarthritis–affected status and visit*

	Year 1		Year 10		Year 15	
	Unaffected	Affected	Unaffected	Affected	Unaffected	Affected
IL-6						
Year 5						
*P* versus unaffected		0.0002		0.0002		0.003
No. of subjects	370	57	257	169	154	165
Mean ± SD pg/ml	0.05 ± 0.88	0.55 ± 1.11	−0.02 ± 0.88	0.32 ± 0.97	−0.03 ± 0.83	0.27 ± 0.98
Year 8						
*P* versus unaffected		0.032†		0.037†		0.264
No. of subjects	412	58	290	180	176	176
Mean ± SD pg/ml	0.56 ± 0.91	0.83 ± 0.79	0.52 ± 0.94	0.70 ± 0.82	0.48 ± 0.95	0.59 ± 0.86
Year 15						
*P* versus unaffected		0.127		0.026†		0.011†
No. of subjects	283	39	200	122	156	156
Mean ± SD pg/ml	0.27 ± 0.78	0.48 ± 0.83	0.22 ± 0.77	0.42 ± 0.81	0.16 ± 0.77	0.39 ± 0.78
TNFα						
Year 5						
*P* versus unaffected		0.832		0.285		0.3775
No. of subjects	371	56	258	169	155	165
Mean ± SD pg/ml	1.37 ± 1.06	1.41 ± 1.05	1.33 ± 1.06	1.45 ± 1.06	1.34 ± 1.06	1.44 ± 1.13
Year 8						
*P* versus unaffected		0.0007†		0.375		0.597
No. of subjects	412	58	290	180	176	176
Mean ± SD pg/ml	0.79 ± 0.75	1.16 ± 0.87	0.81 ± 0.78	0.88 ± 0.76	0.82 ± 0.81	0.86 ± 0.75
Year 15						
*P* versus unaffected		0.458		0.834		0.656
No. of subjects	283	39	200	122	157	156
Mean ± SD pg/ml	0.28 ± 0.50	0.22 ± 0.44	0.27 ± 0.50	0.28 ± 0.47	0.29 ± 0.51	0.26 ± 0.47
CRP						
Year 5						
*P* versus unaffected		0.006†		0.010†		0.096
No. of subjects	358	56	248	166	146	163
Mean ± SD mg/liter	−1.82 ± 1.08	−1.45 ± 1.15	−1.93 ± 1.02	−1.65 ± 1.15	−1.94 ± 1.01	−1.74 ± 1.14
Year 8						
*P* versus unaffected		0.051†		0.555		0.851
No. of subjects	409	62	287	184	174	174
Mean ± SD mg/liter	−2.01 ± 0.99	−1.75 ± 0.94	−2.00 ± 1.02	−1.94 ± 0.94	−1.99 ± 1.02	−1.97 ± 0.93
Year 15						
*P* versus unaffected		0.377		0.026†		0.001†
No. of subjects	280	39	198	121	154	155
Mean ± SD mg/liter	−1.70 ± 0.99	−1.55 ± 0.93	−1.77 ± 0.97	−1.52 ± 0.98	−1.86 ± 0.99	−1.51 ± 0.94

* IL-6 = interleukin-6; TNFα = tumor necrosis factor α; CRP = C-reactive protein.

† *P* < 0.05 versus unaffected, by analysis of variance.

BMI, IL-6 levels, and CRP levels, as well as the mean age per time point, were then examined by multiple logistic regression analysis. As shown in Table 3, age and BMI at baseline and IL-6 levels at year 5 were retained in the model as significant predictors of subsequent RKOA at year 10, suggesting that individuals were more likely to be diagnosed as having RKOA if they had a higher BMI at baseline (OR 1.115, 95% CI 1.051–1.184 per kg/m^2^) and increased IL-6 levels (logarithm-transformed) at year 5 (OR 1.340, 95% CI 1.064–1.687 pg/ml).

**Table 3:** Multiple logistic regression analysis of significant predictors of radiographic knee osteoarthritis at year 10*

Effect	Estimate	Standard error	Odds ratio (unit change)	95% CI	*P*
Intercept	−7.520	1.254	–		4.5 × 10^−9^
Age	0.076	0.018	1.079	1.041–1.119	3.93 × 10^−5^
BMI	0.109	0.109	1.115	1.051–1.184	0.0003
Ln_IL-6	0.293	0.117	1.340	1.064–1.687	0.0129

* Independent variables included age and body mass index (BMI) at baseline and C-reactive protein and interleukin-6 (IL-6) levels at year 5. Only significant results are shown. 95% CI = 95% confidence interval; Ln_IL-6 = natural logarithm–transformed IL-6 levels.

Finally, longitudinal modeling of repeated measurements using mixed-effects logistic regression analysis confirmed the results obtained by multiple logistic regression (Table 4). In the longitudinal analysis, for the sake of simplicity, determinations of RKOA, age, and BMI at years 1, 10, and 15 and the cytokine measurements at years 5, 8, and 15 were designated visits 1, 2, and 3, respectively. Results were consistent for diverse comparisons of the visits, e.g., visit 1 compared with visit 2, visit 1 compared with visit 3, or all 3 visits compared. The results showed that older age, higher BMI, and increased IL-6 levels were all significant independent predictors of RKOA status, while CRP levels did not make a significant independent contribution.

**Table 4:** Mixed-effects logistic regression analysis of the significant predictors of radiographic knee osteoarthritis from repeated measurements at years 1, 10, and 15*

	Visits 1 and 2			Visits 1 and 3			Visits 1, 2, and 3		
Effect	Estimate	Standard error	*P*	Estimate	Standard error	*P*	Estimate	Standard error	*P*
Intercept	−28.988	7.140	0.001	−55.797	14.114	<0.001	−84.624	13.874	<0.001
Age	0.289	0.076	<0.001	0.518	0.137	<0.001	0.917	0.163	<0.001
BMI	0.325	0.912	<0.001	0.727	0.211	0.001	0.765	0.165	<0.001
Ln_IL-6	0.137	0.063	0.028	1.327	0.625	0.034	1.107	0.490	0.024

* Independent variables included age and BMI at years 1, 10, and 15 and C-reactive protein and IL-6 levels at years 5, 8, and 15 (designated visits 1, 2, and 3, respectively). Only significant results are shown. See Table 3 for definitions.

**Figure 2:**
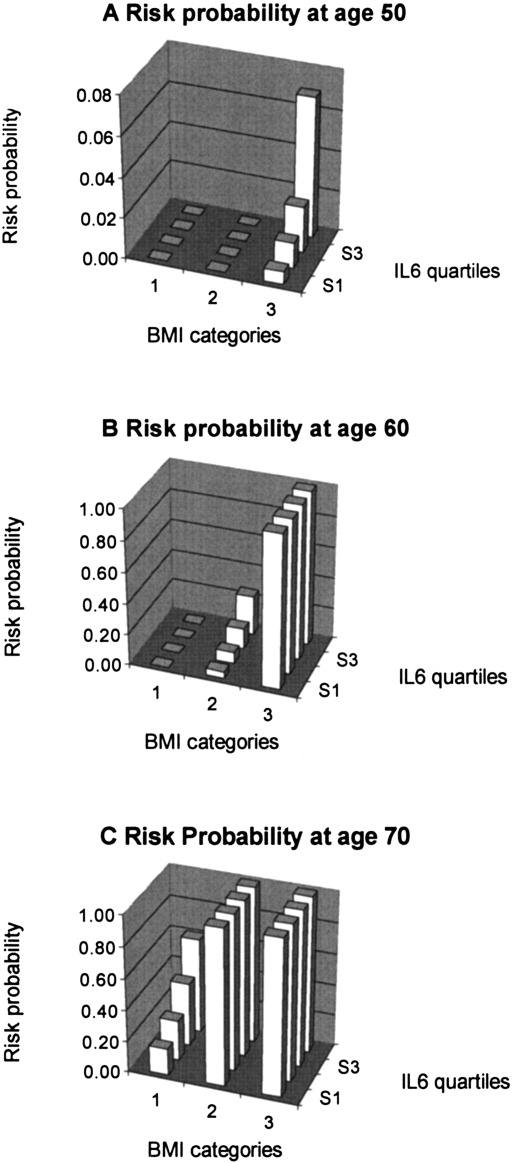
Risk probability for radiographic knee osteoarthritis (Kellgren/Lawrence grade ≥2) in the Chingford Study at ages 50 years (**A**), 60 years (**B**), and 70 years (**C**) as a function of body mass index (BMI) and circulating levels of interleukin-6 (IL-6). Values are the mixed-effects logistic model parameter estimates (see Table 4) for 3 categories of BMI (1 = 21–30 kg/m^2^, 2 = 31–40 kg/m^2^, and 3 = 41–50 kg/m^2^) and for quartiles of IL-6 distribution (S1– S4). The values of the boundary at the 25th, 50th (median), and 75th percentiles of IL-6 levels were 0.767 pg/ml, 1.235 pg/ml, and 2.310 pg/ml, respectively, with corresponding minimum and maximum values of 0.162 pg/ml and 30.228 pg/ml, respectively, at the tails of the distribution. All IL-6 data were logarithm-transformed.

Figure 2 shows the predicted risk of RKOA at ages 50, 60, and 70 years as a function of BMI and IL-6 levels; i.e., we estimated the probability of the individual having RKOA according to 3 specific categories of BMI (1 = 21–30 kg/m^2^, 2 = 31–40 kg/m^2^, and 3 = 41–50 kg/m^2^) and according to quartiles of logarithm-transformed mean IL-6 measurements. The interplay observed between IL-6 levels and BMI indicated that at age 50 years, the effect of both BMI and IL-6 levels appeared in only very-obese individuals (those in BMI category 3) (Figure 2A). At age 70 years, the effect was evident in less-obese individuals (those with a BMI of ≤30 kg/m^2^) (Figure 2C). The effect of IL-6 levels was seen particularly well in this latter group (Figure 2C) and also in individuals at age 50 years whose BMI was ≤50 kg/m^2^ (Figure 2A). The risk of RKOA clearly increased with increasing IL-6 levels in individuals at age 60 years whose BMI was ≤40 kg/m^2^ (Figure 2B).

When the effects of IL-6 levels alone were assessed, the comparison of the quartiles showed that the likelihood of being diagnosed as having RKOA in individuals with IL-6 levels in the fourth quartile (versus the first quartile) was 2.74 (95% CI 1.94–3.87; *P* < 0.001) when only individuals with a K/L grade ≥2 were considered. In individuals with severe RKOA (K/L grade >2 in either or both knees), the OR for those in the fourth quartile of IL-6 levels (versus the first quartile) reached 3.59 (95% CI 1.93–6.68; *P* < 0.001).

## DISCUSSION

The prevalence of RKOA in this population sample of women whose data were obtained from general practice registers in the UK increased during the 15 years of followup, from 14.7% to 48.7%. We also noted the higher prevalence of OA in the right knee (versus the left), suggesting involvement of mechanical factors in RKOA etiologic development. The novel finding of this study was a consistent association of IL-6 circulating levels with the prevalence and incidence of RKOA as assessed by K/L grade. IL-6 levels were consistently found to be associated with the RKOA prevalence in the specific year and when the 3 visits were combined. Two other variables that were consistent predictors of the appearance of RKOA were age and BMI (Tables 3 and 4).

Although the evidence for an association with serum TNFα levels was negligible, CRP levels were also significantly associated with RKOA when considered separately (Table 2). However, the effect of CRP disappeared after adjustment for age and IL-6 levels in multivariate analysis (Tables 3 and 4), likely because the CRP concentration and the levels of IL-6 are related biologically. In our study, the serum concentrations of CRP and IL-6 showed a significant correlation, which varied between 0.22 and 0.48 (*P* < 0.001), depending on the year of the visit. CRP levels showed very low, nonsignificant correlations with age. This may account for our earlier findings of a significant predictive effect of CRP in the same sample (16). In the present sample, when the IL-6 effect was considered alone, it showed a highly significant association with RKOA (*P* < 0.001). As a result, the prevalence comparison between the first and fourth quartiles of IL-6 levels yielded an OR of 2.74 (95% CI 1.94–3.87; *P* < 0.001) for those with K/L grade ≥2 knees, and an OR of 3.59 (95% CI 1.93–6.68; *P* < 0.001) when only those with severe RKOA (K/L grade >2) were considered.

A growing body of evidence suggests that development of OA, even at the early stages, is often accompanied by inflammation (24). This is consistent with the findings from epidemiologic studies in which the severity and progression of tibiofemoral cartilage damage were observed to be more common and severe in patients having reactive and inflamed synovial fluid (25). Moreover, elevated levels of cytokines such as IL-1β and TNFα, both of which are regulators of inflammation, are found in the disease process, although their precise cell of origin is not clear (17, 26). Increased levels of CRP have been associated with the prevalence and progression of knee and hip OA in several studies (16, 18, 27), although in other studies, this association became nonsignificant after adjustment for BMI (19). Of note, however, to our knowledge, there is no published study examining the association between OA and circulating IL-6 levels. Thus, the present study is the first to assess this association.

One potential source of these inflammatory cytokines is adipose tissue, which accompanies obesity (28) and which is known to be associated with high levels of biomarkers of inflammation, including IL-6 (7, 29). In the study by Das (29), the results suggest that synthesis of TNFα (and leptin) in adipose tissue could induce the production of IL-6 and the acute-phase reactant CRP. Increased levels of these cytokines may, in turn, contribute to the development of OA. Our data support this observation. In our study, we observed significantly higher BMI values in individuals affected with RKOA. Moreover, the correlation between BMI and RKOA was found not only with the weight-bearing knee joints but also with hand OA in the present sample (results not shown). The link between obesity and hand OA was noted some time ago (e.g., see ref.^30^). Although the mechanism remains to be debated, one of the most popular theories is that fat tissue causes systemic release of proinflammatory cytokines. Circulating IL-6, as mentioned above, is known to stimulate CRP production by the liver. Indeed, we observed consistent correlations between the CRP levels and IL-6 levels, and significantly higher CRP levels in RKOA-affected individuals.

There are several limitations to this study. First, there is a discrepancy in the timing of the study assessments of RKOA and the cytokine assays (Table 1). Serum samples were not available at baseline when the first set of radiographs was obtained. Thus, it is not known how many individuals had RKOA at year 5 (when the first serum samples were assayed). Likewise, the number of subjects having elevated IL-6 levels at baseline, when the first radiographic examination was conducted, is not known. This was the main reason for testing the association between the cytokine levels at year 5 with RKOA at year 10.

The second limitation is that inflammatory cytokines were measured in the circulation and not in synovial fluid. It is possible that serum cytokines could originate from a different tissue. In addition, the decreasing sample size with each followup visit, albeit inevitable in a longitudinal study of this kind, did contribute to a loss of statistical power. We found, however, no evidence of a systematic bias resulting from the loss of baseline subjects at followup. First, only a relatively small proportion of the sample was lost during the followup period. Second, IL-6 levels and OA prevalence rates at the baseline examination did not differ between those who subsequently dropped out and the total group.

The effect of limited sample size was pronounced when multivariate analyses were undertaken, since the small sample numbers at baseline would not allow us to specifically examine progressive disease. However, we believe that incidence and progression may be interchangeable in this kind of study. For example, since we defined RKOA as a K/L grade ≥2 in either knee, those with a K/L grade ≥1 at baseline whose disease then progressed would be considered an incident case, although really they should be considered “progressors.” Indeed, when we compared IL-6 levels according to K/L grade (unaffected = K/L grade <2, minor RKOA = K/L grade = 2, and severe RKOA = K/L grade >2), a consistent association of continuous increases in these parameters was observed (Figure 1), which was consistent with the ORs for RKOA by quartiles of IL-6 levels. Similarly, if we had used magnetic resonance imaging or another more sensitive measure of disease, then most incident cases would probably be those with minor OA detectable at baseline. Clearly, the study would have benefited from current radiologic standards and techniques for the knee, although any misclassification due to the extended knee position would have acted to obscure the results.

It is also important to note that this study did not examine the effect of OA comorbidity in the other joints. This would lead to a much more complex analysis of the data, which was beyond the scope of this study.

A further, minor limitation is that the study was undertaken using data only from female subjects. OA is increasingly recognized to be site- and sex-specific, and therefore the present results may not be directly extrapolated to men. There is little evidence, however, to suggest that cytokine expression differs significantly between the sexes.

In conclusion, the present findings suggest that a higher BMI and increased circulating levels of IL-6 are associated with the development of RKOA. IL-6 is therefore a potential new biomarker for OA development and may provide useful information in the prediction of disease outcome. Moreover, highlighting the potential key role of IL-6 in the disease process may lead to studies of IL-6 blockade as a therapeutic strategy in OA.

## AUTHOR CONTRIBUTIONS

All authors were involved in drafting the article or revising it critically for important intellectual content, and all authors approved the final version to be published. Dr. Spector had full access to all of the data in the study and takes responsibility for the integrity of the data and the accuracy of the data analysis.

**Study conception and design.** Livshits, Zhai, Spector.

**Acquisition of data.** Wang, Spector.

**Analysis and interpretation of data.** Livshits, Hart, Kato, Williams.
